# PP56 Frequency Of Indirect Comparisons In Technology Assessment For Incorporation In The Brazilian Supplementary Health System: Meta-Research

**DOI:** 10.1017/S0266462325102754

**Published:** 2025-12-29

**Authors:** Camila Cruz, Ana Luiza C. Martimbianco, Carolina de O. C. Latorraca, Isabela P. de Toledo, Roberta B. Silva, Cecilia Menezes Farinasso, Patrícia do C. S. Parreira, Verônica Colpani, Rafael L. Pacheco, Rachel Riera

## Abstract

**Introduction:**

For the technology incorporating process, indirect comparisons (IC) may be presented as alternatives when head-to-head studies are unavailable. However, health technology assessment (HTA) reports often fail to address the methodological fragilities of network meta-analysis. This interim analysis examined the frequency of IC methods in technology assessments for incorporation into the Brazilian supplementary health system and inclusion in the National Agency of Supplementary Health (ANS) catalog.

**Methods:**

This meta-research study was conducted at the Center of HTA, Hospital Sírio-Libanês, São Paulo, Brazil. A search on the ANS website identified critical analysis reports of technologies incorporated by ANS in 2023 and 2024. Eligible reports were analyzed to identify the frequency of IC in plaintiff’s submissions and in ANS analyses. The incremental budget impact of incorporating technologies with IC in HTA reports was also evaluated.

**Results:**

A total of 47 reports were evaluated. Of these, 36.2 percent of the submissions and 25.5 percent of the ANS analyses included IC, mostly using network meta-analysis. Among 32 favorable recommendations, IC appeared in 31.3 percent of submissions and 25 percent of ANS analyses. From 2023 to 2024, the use of IC increased by 12.7 percent in submissions and by 21.8 percent in ANS analyses. Within incorporated technologies containing IC, the incremental budget impact analysis predicted savings of BRL61,385,084.02 to BRL174,217,905.44 for savings scenarios, and increased spending of BRL179,524,075.54 to BRL488,084,349.61 for increased investment scenarios.

**Conclusions:**

IC methods were present in approximately 25 to 30 percent of all technology assessments for incorporation into the Brazilian supplementary health system, with an increased number observed from 2023 to 2024. Within technology assessments employing IC, projected financial outcomes indicated savings in some scenarios, while others suggested substantial increases in budget expenditure.

